# Retrograde transvenous thoracic duct embolization for lymphatic leakage after retroperitoneal tumor and lymph node resection: a case report and literature review

**DOI:** 10.1186/s40792-024-01856-3

**Published:** 2024-03-08

**Authors:** Go Kinoshita, Koichi Morisaki, Daisuke Okamoto, Takehiko Aoyagi, Shinichiro Yoshino, Kentaro Inoue, Tomoharu Yoshizumi

**Affiliations:** 1 Department of Surgery and Science, Graduate School of Medical Sciences, Kyushu University, 3-1-1, Maidashi, Higashi-Ku, Fukuoka, 812-8582 Japan; 2 Department of Clinical Radiology, Graduate School of Medical Sciences, Kyushu University Hospital, 3-1-1, Maidashi, Higashi-Ku, Fukuoka, 812-8582 Japan

**Keywords:** Lymphatic leakage, Retrograde transvenous thoracic duct embolization, Chylothorax

## Abstract

**Background:**

Postoperative lymphatic leakage is a complication of ineffective conservative treatment for retroperitoneal mass. Herein, we report a case of lymphatic leakage that arose after retroperitoneal tumor resection and that was treated with retrograde transvenous thoracic duct embolization.

**Case presentation:**

A 28-year-old man with persistent abdominal pain was diagnosed with a large retroperitoneal metastatic tumor measuring 10 cm and a subdiaphragmatic lymph node originating from a testicular tumor. After high orchidectomy and neoadjuvant chemotherapy, the subdiaphragmatic lymph node and retroperitoneal tumor were resected together with the abdominal aorta; the latter was reconstructed using a prosthetic graft. Postoperatively, the patient developed chylothorax. No improvement was observed after conservative treatment that included fasting and somatostatin therapy. The leakage site could not be identified using antegrade lymphangiography of the bilateral inguinal lymph nodes, but was detected using retrograde transvenous lymphangiography. The leakage site was successfully embolized.

**Conclusion:**

This case report describes successful treatment with retrograde transvenous thoracic duct embolization for chylothorax following resection of a retroperitoneal tumor and lymph node. This approach is a less invasive and more effective mode of treatment for chylothorax and should be considered before surgical thoracic duct ligation when the leakage point cannot be identified using the antegrade approach.

## Background

Postoperative lymphatic leakage is a relatively rare complication, occurring in 3% of postoperative esophageal cancers [1], 2–7% of retroperitoneal lymph node resections, and less than 1% of aortic aneurysm repairs [2]. Treatment for lymphatic leakage includes conservative management, somatostatin therapy, antegrade and retrograde thoracic duct embolization, percutaneous transabdominal thoracic duct embolization, or surgical thoracic duct ligation [3]. The first choice is conservative treatment with fasting and somatostatin therapy placement of a drainage duct. However, when the volume of lymphatic fluid exceeds 1000 mL/day, conservative treatment alone is unlikely to cure the leakage [4]. If conservative treatment is unsuccessful, invasive treatment may be administered that includes lymphangiography with thoracic duct embolization, transabdominal embolization, and surgical thoracic duct ligation [1, 5, 6]. With respect to lymphangiography, two approaches have been reported: the antegrade approach from the inguinal lymph nodes and the retrograde transvenous approach. An antegrade approach is usually selected, but its success rate is only 70% [7]. The retrograde transvenous approach was first reported by Mittleider et al. in 2008 [1]. Recently, Jun et al. reported that the retrograde approach combined with the antegrade approach may improve the treatment outcome of thoracic duct embolization [7]. We report a case of chylothorax after retroperitoneal tumor resection treated using the antegrade approach via the inguinal lymph node and retrograde transvenous thoracic duct embolization.

## Case presentation

**Fig. 1:**
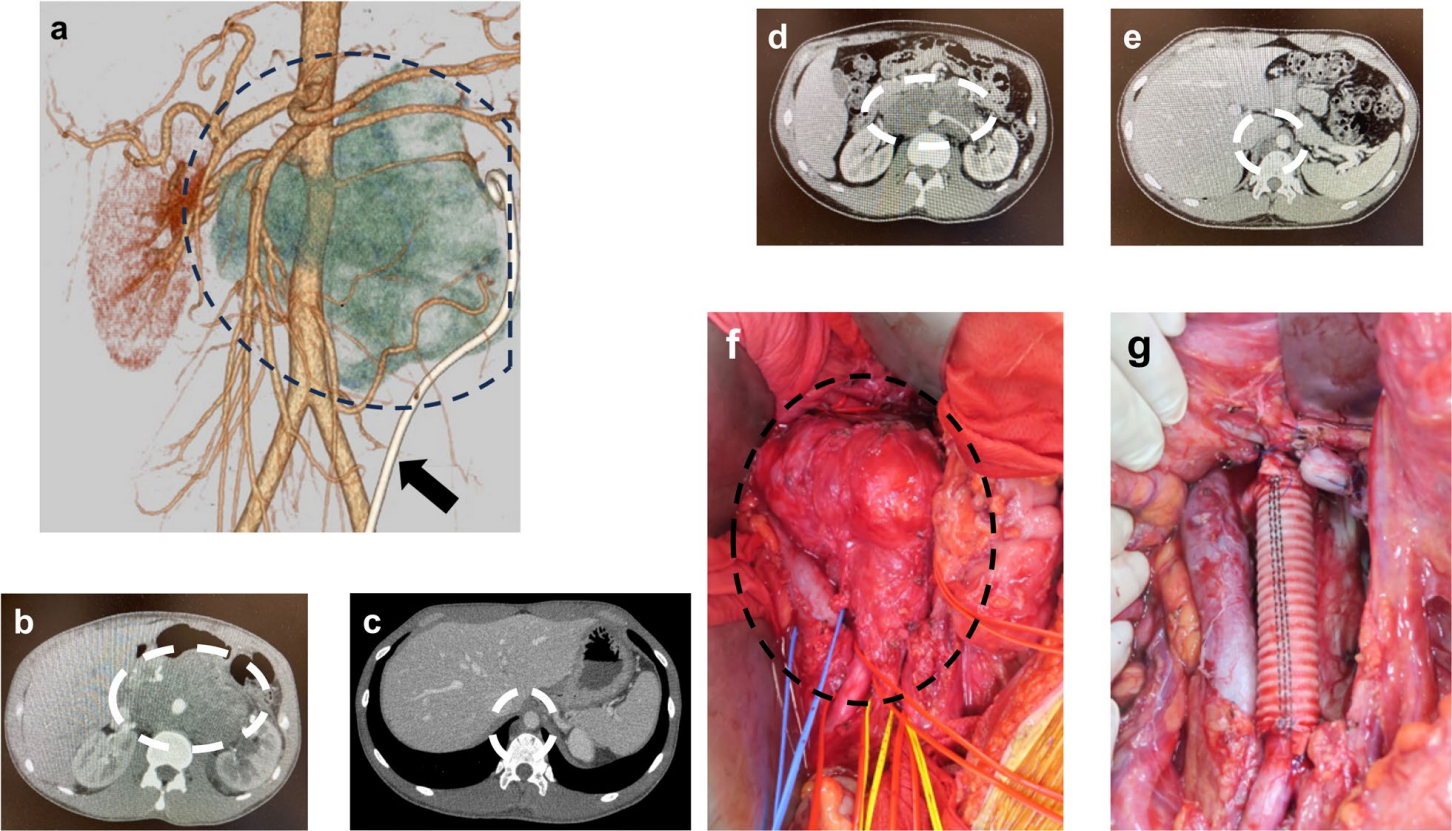
Computed tomography (CT) images and intraoperative findings. **a** The three-dimensional CT image captured before chemotherapy. The dotted line delineates the extent of the tumor. The black arrow indicates the ureteral stent. **b, c** CT images before chemotherapy. **d, e** CT images after chemotherapy. **f** Intraoperative findings. The dotted line indicates the extent of the tumor. **g** Intraoperative findings after tumor resection with abdominal aorta and reconstruction by prosthetic graft

**Fig. 2:**
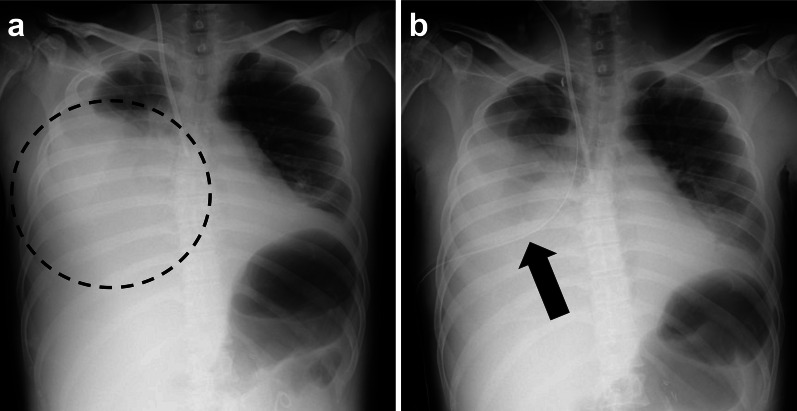
Chest radiograph images. **a** Chest radiograph captured on postoperative day 4. Large pleural effusion is visible within the area delineated by the dotted line. **b** After thoracic drain placement; the black arrow indicates the drain

A 28-year-old man with persistent abdominal pain was examined and found to have a large retroperitoneal tumor measuring 10 cm diameter and a testicular tumor. A retroperitoneal tumor involving the superior mesenteric artery, bilateral renal arteries, and abdominal aorta, and an enlarged subdiaphragmatic lymph node were revealed on computed tomography (Fig. 1a–c). In accordance with the guidelines for the treatment of testicular tumors, the patient underwent high orchidectomy. Histological examination showed that the tumor was a seminoma. After chemotherapy—four courses of bleomycin, etoposide, and platinum therapy; two courses of etoposide and cisplatin therapy; and two courses of paclitaxel, ifosfamide, and cisplatin therapy—and shrinkage of the tumor (Fig. 1d, e), the subdiaphragmatic lymph node and retroperitoneal tumor with the abdominal aorta were resected. The abdominal aorta was resected from infrarenal aorta to terminal aorta and reconstructed with a prosthetic graft (Fig. 1f, g). On the fourth day after surgery, a chest radiograph showed massive pleural effusion (Fig. 2a) and a drainage tube was inserted into the patient’s right chest cavity (Fig. 2b). When the patient resumed food intake on the seventh day after surgery, the pleural effusion changed from serous to chyle, and the patient was diagnosed with lymphatic leakage. The patient was administered conservative treatment consisting of fasting and somatostatin therapy (300 µg/day); however, the volume of fluid drained continued at approximately 1000 mL/day.

**Fig. 3:**
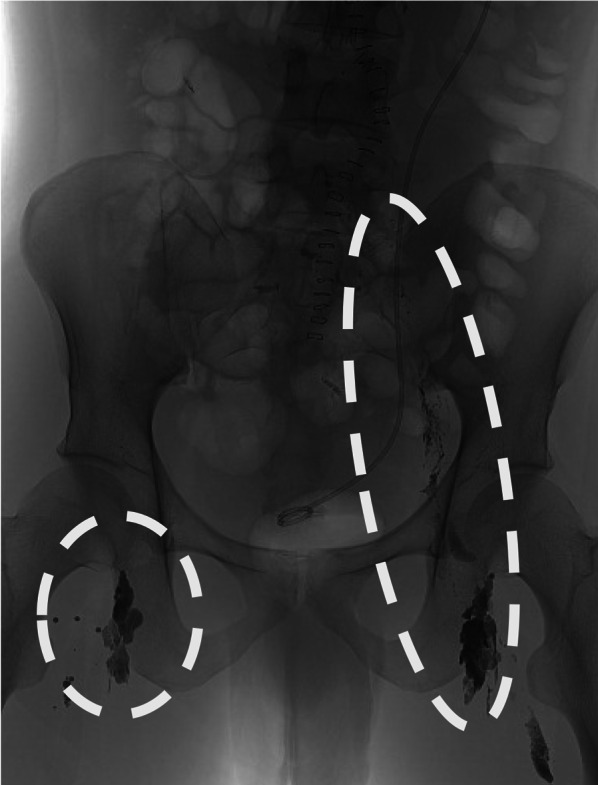
Contrast image after the antegrade lymphangiography. The circles show the reach of Lipiodol contrast. Lipiodol contrast from the right inguinal lymph nodes flowed into the vein. Lipiodol contrast from the left inguinal lymph nodes reached near the left kidney

**Fig. 4:**
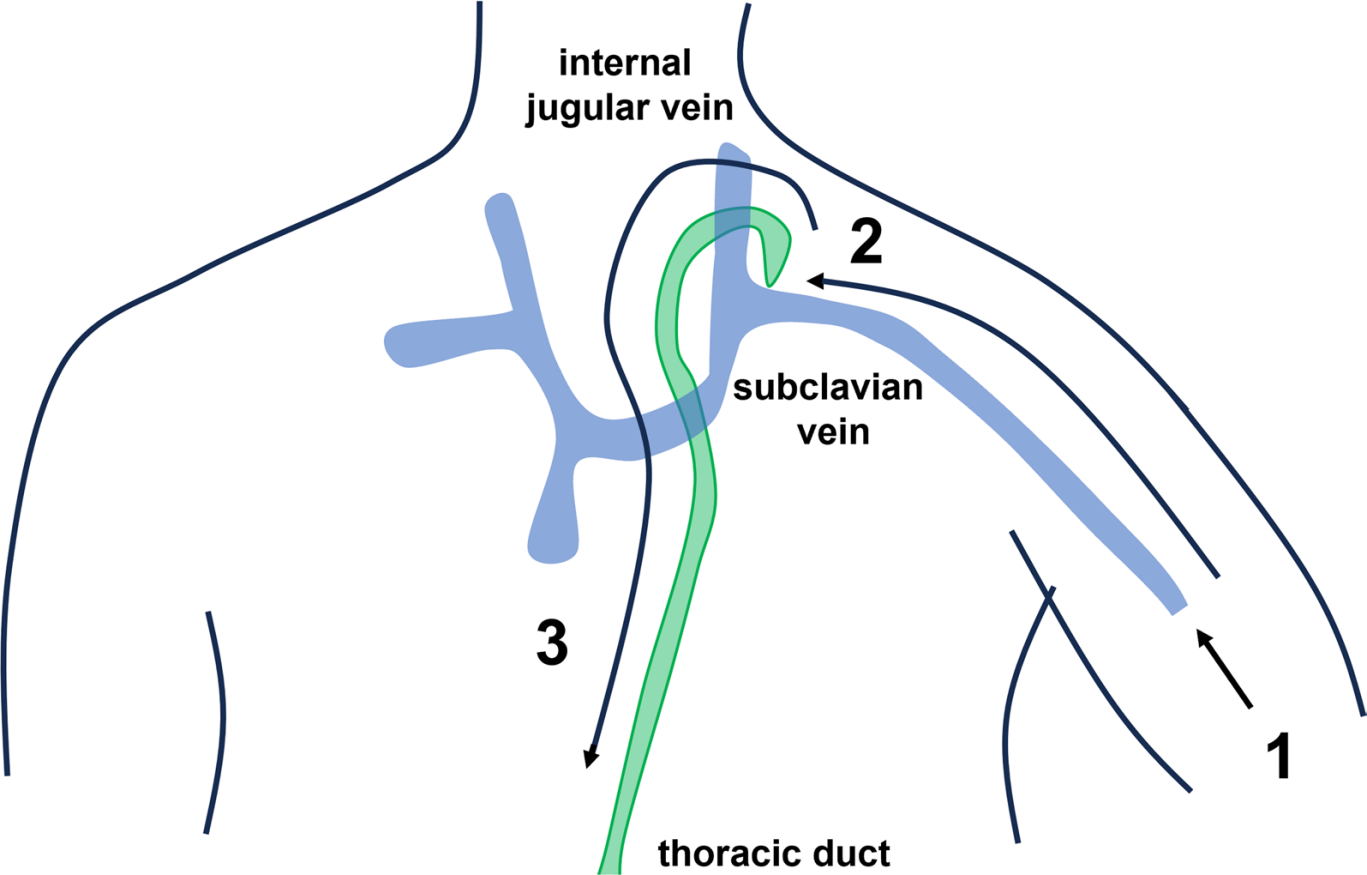
Schema of the retrograde transvenous approach. 1. Puncture the arm vein and place a sheath. 2. Advance the guidewire and catheter and cannulate the entrance of the thoracic duct. 3. Advance the guidewire and catheter into the thoracic duct

**Fig. 5:**
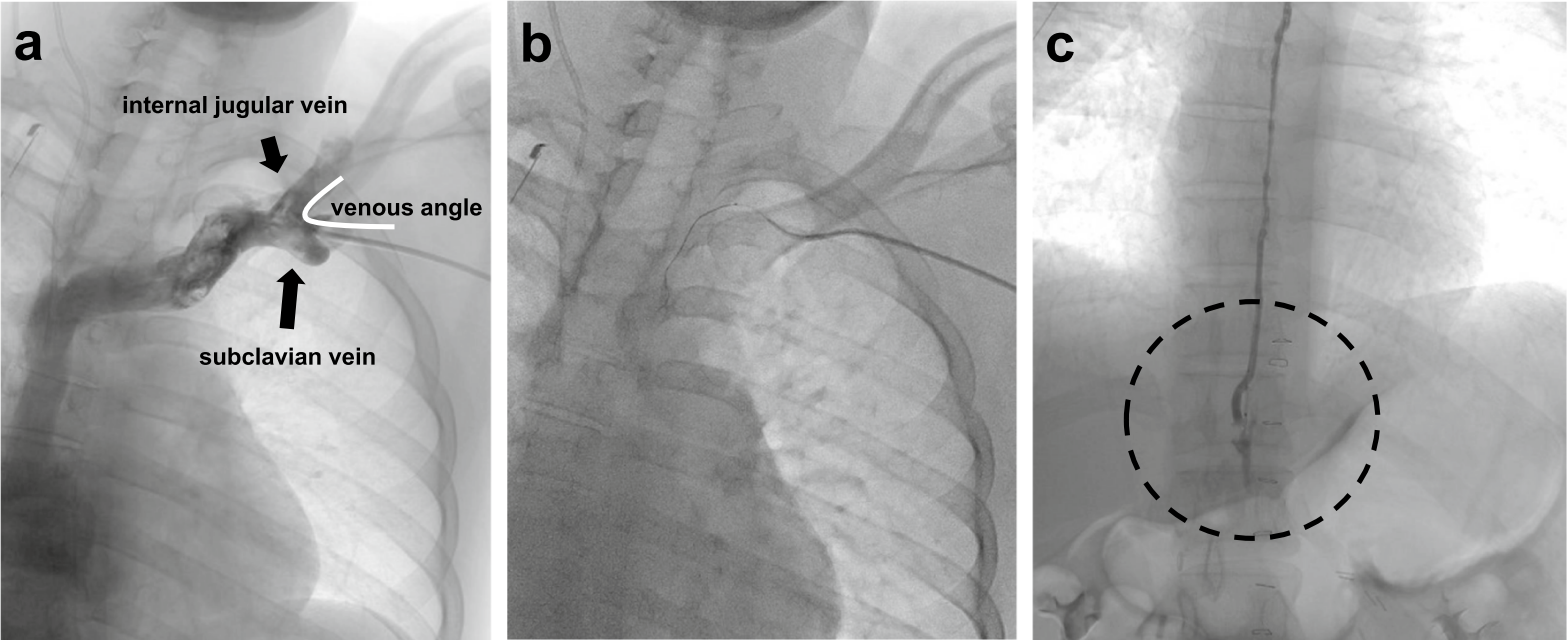
Contrast images. **a** Black arrows indicate the left internal jugular and subclavian veins. The angle created by the two veins is the venous angle. **b** The wire and catheter are advancing into the thoracic duct. **c** Contrast image of the thoracic duct. The thoracic duct is disrupted at the dotted line

On the thirteenth postoperative day, antegrade lymphangiography of the bilateral inguinal lymph nodes was performed; however, no lymphatic leakage was identified (Fig. 3). On the fifteenth day after surgery, a lymphangiography using a retrograde transvenous approach was performed. The procedure of retrograde transvenous approach was shown in the schema (Fig. 4). This schema was created based on the report by Sato et al. [8]. The left cephalic vein was percutaneously punctured and an 11-cm 7 Fr sheath was placed. A 7 Fr 65-cm KMP catheter was advanced to the venous angle using a 0.035-in. 180-cm Radifocus guidewire (Terumo, Tokyo, Japan) (Fig. 5a). Cannulation of the thoracic duct was performed using a 0.014-in. 180-cm Asahi Chikai V guidewire (ASAHI INTECC, Aichi, Japan) supported by a 2.0/2.4 Fr 150-cm Leonis Mova catheter (SB-KAWASUMI LABORATORIES, Kanagawa, Japan) (Fig. 5b). The catheter was advanced to the eleventh thoracic vertebra level through several thoracic duct valves. Lymphangiography revealed interruption of the thoracic duct and spillage of contrast with Lipiodol outside the duct (Fig. 5c). The thoracic duct was embolized with 0.4 mL of a 1:2 mixture of n-butyl-2-cyanoacrylate and Lipiodol. After thoracic duct embolization, the drainage volume decreased to 100 mL/day, and the drainage tube was removed. The patient was discharged on the twenty-ninth day after surgery without recurrent effusions.

## Discussion

In the present case, the postoperative chylothorax was successfully treated with retrograde transvenous thoracic duct embolization after the antegrade approach failed. The failure of the antegrade approach in this case may have occurred because the distal side of the thoracic duct was tightly ligated during resection of the subdiaphragmatic lymph node. As demonstrated in this case, the retrograde transvenous approach is an effective and less invasive treatment than the percutaneous transthoracic or abdominal approach, or surgical thoracic duct ligation.

With respect to the transthoracic or abdominal approaches, Itkin et al. reported a success rate for embolization of 68.9% (73/106 cases), a clinical success rate of 81.7% (58/71 cases), and a complication rate of 2.8% (3/71 cases) [3]. Pamarthi et al. reported a success rate for embolization of 79.2% (95/120 cases), a clinical success rate of 50.8% (61/120 cases), and a complication rate of 5.8% (7/120 cases) [4]. The most common complication associated with this procedure was influx of embolic material into the pulmonary artery, which did not require additional treatment [3, 4]. In cases where lymph nodes have been extensively resected, the problem that arises is that transabdominal puncture cannot be performed because the flow of lymph fluid is obstructed and the site of the thoracic duct injury cannot be identified using lymphangiography [5]. Furthermore, the transabdominal approach carries the risk of organ penetration, including the arteries, biliary system, and intestines.

Antegrade thoracic duct embolization is performed by puncturing the inguinal lymph node under ultrasound guidance [3]. The technical success rate of the antegrade approach is approximately 70% [3, 4, 7]. As in the present case, Jun et al. showed that using a retrograde transvenous approach after failure of the antegrade approach could improve the technical success [7]. The retrograde transvenous approach was first reported by Mittleider et al. in 2008 [1].

We conducted a review of studies in which the retrograde transvenous approach was used and a summary of the findings is presented in Table 1. We reviewed the literature published between 2008 and September 2023, and retrieved the details of only 58 cases reported in English by 18 authors. In the reports retrieved, the success rates of cannulation and lymphatic leakage embolization were 73.6% (39/53 cases) and 80% (32/40 cases), respectively. The clinical success rate was 75.0% (27/36 cases) and the complication rate was 2.8% (1/36 cases).

**Table 1:** Cases in literature reporting retrograde transvenous thoracic duct embolization

Authors	Year	Number of patients	Age	Sex	Causes of lymph leakage	Technical success	Embolization success	Clinical success	Complications
Mittleider et al. [1]	2008	1	60 s	M:1	Postoperative pancreatic cancer:1	1/1	1/1	0/1	None
Koike et al. [9]	2013	2	50-60 s	M:1, F:1	Postoperative thoracic aortic aneurysm:1 spinal arteriovenous malformation:1	2/2	2/2	1/2	None
Schild et al. [10]	2015	1	–	–	–	1/1	1/1	1/1	None
Sugimura et al. [6]	2017	1	60 s	M:1	Liver cirrhosis:1	1/1	1/1	1/1	None
Arslan et al. [11]	2017	1	50 s	F:1	Cervical lymph node dissection:1	1/1	1/1	1/1	None
Hussain et al. [12]	2018	1	20 s	M:1	Traffic trauma:1	1/1	1/1	1/1	None
Srinivasa et al. [2]	2018	3	10-70 s	M:2, F:1	Post-nephrectomy:2, hypoplastic left heart:1	3/3	3/3	3/3	None
Majdalany et al. [13]	2018	3	60-70 s	M:2, F:1	Post-nephrectomy:2,post-pancreatectomy and splenectomy	3/3	3/3	2/3	None
Kariya et al. [14]	2018	13	20-80 s	M:5, F:8	Lymphangiectasis:3, thyroid cancer:1, systemic lupus erythematosus:1, acquired immunodeficiency syndrome:1, past esophageal cancer chemoradiation:1, postoperative esophageal cancer:1, gastric cancer surgery:2, lung cancer:1, cervical cancer:1, unknown:1	8/13	1/1	1/1	Thoracic valve injury
Rott et al. [15]	2020	1	60 s	F:1	Postoperative Nissen surgery:1	1/1	1/1	1/1	None
Morikawa et al. [5]	2020	3	40-70 s	M:1, F:2	Postoperative paraaortic lymph node dissection, post-nephrectomy:2	3/3	3/3	3/3	None
Dar et al. [16]	2021	1	50 s	M:1	Traffic trauma:1	1/1	1/1	1/1	None
Sato et al. [8]	2021	1	70 s	M:1	Postoperative esophageal cancer:1	1/1	1/1	1/1	None
Kalia et al. [17]	2022	2	50 s	F:2	Post-nephrectomy:2	1/1	1/1	1/1	None
Seth et al. [18]	2022	1	50 s	M:1	Unknown:1	1/1	1/1	1/1	None
Jun et al. [7]	2022	6	–	–	–	–	–	–	–
Petrini et al. [19]	2023	1	60 s	M:1	Non-Hodgkin lymphoma:1	1/1	1/1	1/1	None
Kim et al. [20]	2023	16	–	–	–	8/16	8/16	6/12	–

The retrograde transvenous approach is a less invasive and more effective treatment for chylothorax; however, it may be considered technically more challenging. The end of the thoracic duct has an ostial valve, which is usually difficult to visualize, and the confluence of the subclavian vein and the thoracic duct is difficult to cannulate because of the variety of veins. Furthermore, valves in the thoracic duct and variations in the thoracic duct may prevent the contrast agent from reaching the injured area. When a retrograde venous approach is used, lymphangiography from the inguinal lymph nodes should be used to identify the entry site of the thoracic duct [5]. If lymphangiography from the inguinal lymph node is not available, lymphangiography should be performed beforehand using magnetic resonance imaging to determine the location around the thoracic duct and the vein. Balloon-occluded retrograde lymphangiography may also be useful [12]. A 5 Fr balloon catheter is inserted into the thoracic duct, and a contrast agent is released from the tip of the balloon catheter with balloon inflation for contrast. By blocking lymphatic drainage, the lower thoracic duct may be contrasted and the site of injury identified.

Another factor that complicates the retrograde transvenous approach is the presence of several patterns in which the thoracic duct may enter the venous angle. The cervical part of the thoracic duct is defined as the distance from the beginning of the thoracic duct to the level of the left brachiocephalic vein, whereas the thoracic part is defined as the remaining section of the thoracic duct [14]. At the cervical part, the plexiform type is defined as having a plexiform configuration without a prominent main duct. In the case of the plexiform type, advancing the catheter to the thoracic duct is difficult, although cannulation to the thoracic entrance was possible in all three cases [14]. Thus, the retrograde transvenous approach is difficult to perform in cases where the cervical part is of the plexiform type.

## Conclusions

This case report describes successful treatment with retrograde transvenous thoracic duct embolization for chylothorax following the resection of a retroperitoneal tumor and lymph node. This approach is a less invasive and more effective treatment for chylothorax and should be considered before surgical thoracic duct ligation when the leakage site cannot be identified using an antegrade approach.

## Data Availability

Not applicable.

## Abbreviation

CT: Computed tomography
